# Physical Activity Before and During Pregnancy and Neurodevelopment in Early Childhood

**DOI:** 10.1001/jamanetworkopen.2026.0345

**Published:** 2026-03-03

**Authors:** Io Kumasaka, Tomohisa Suzuki, Keita Kanamori, Yuichiro Miura, Chiharu Ota

**Affiliations:** 1 Department of Development and Environmental Medicine, Tohoku University Graduate School of Medicine, Sendai, Japan; 2 Environment and Genome Research Center, Tohoku University Graduate School of Medicine, Sendai, Japan; 3 Department of Feto-Maternal Medical Science, Tohoku University Graduate School of Medicine, Sendai, Japan; 4 Department of Pediatrics, Tohoku University Hospital, Sendai, Japan

## Abstract

**Importance:**

Prenatal maternal exercise is recognized for its benefits to both mother and child. However, research examining how maternal physical activity influences the neurodevelopment of offspring is limited.

**Objective:**

To investigate the association between maternal physical activity before and during pregnancy and neurodevelopmental outcomes in children.

**Design, Setting, and Participants:**

This cohort study analyzed data from the Japan Environment and Children’s Study, a nationwide birth cohort funded by the Ministry of the Environment in Japan. Mother-child pairs were recruited and enrolled between January 24, 2011, and March 31, 2014, with child follow-up from birth to 3 years of age. Of 104 062 records, 65 843 were excluded due to missing data or guideline-based criteria, resulting in 38 219 mother-child pairs included in the analysis. The present analysis was conducted between June 24, 2024, and June 30, 2025.

**Exposures:**

Maternal physical activity levels were assessed before and during pregnancy using the International Physical Activity Questionnaire.

**Main Outcomes and Measures:**

Child neurodevelopment was assessed using the Ages and Stages Questionnaires, Third Edition, at 6-month intervals from 6 months to 3 years of age. Five developmental domains were evaluated: communication, gross and fine motor skills, problem solving, and personal-social. Scores were based on caregiver responses and compared with established cutoff scores.

**Results:**

Among the 38 219 mother-child pairs (maternal mean [SD] age, 31.1 [4.8] years; 19 429 [50.8%] male children), multivariable logistic regression showed that higher maternal physical activity was associated with child neurodevelopment. Higher prepregnancy activity was associated with significantly higher odds for each ASQ-3 domain at 6 months of age and higher midpregnancy activity with high odds for the gross motor (odds ratio [OR], 1.18; 95% CI, 1.06-1.33), fine motor (OR, 1.60; 95% CI, 1.37-1.86), and problem solving (OR, 1.23; 95% CI, 1.10-1.38) domains. Higher prepregnancy activity was associated with higher odds for the problem-solving domain (OR, 1.16; 95% CI, 1.01-1.34) at 3 years of age; there was no association with any other domain. Higher midpregnancy activity was not associated with higher odds of any ASQ domain at 3 years of age.

**Conclusions and Relevance:**

In this cohort study of mother-child pairs, maternal physical activity before and during pregnancy was associated with child neurodevelopment, particularly for motor function between 6 months and 1 year of age. Further investigations are required to find the physiological mechanisms explaining how maternal physical activity affects child neurodevelopment.

## Key Points

**Question:**

How is maternal physical activity before and during pregnancy associated with neurodevelopment in children?

**Findings:**

In this cohort study involving 38 219 mother-child pairs, maternal physical activity before and during pregnancy was associated with neurodevelopmental outcomes in late infancy. Moreover, higher levels of maternal exercise were associated with more favorable neurodevelopmental outcomes.

**Meaning:**

These findings suggest that maternal physical activity before and during pregnancy may be associated with optimized early neurodevelopment in offspring, particularly regarding motor function, highlighting the potential benefits of prenatal exercise beyond maternal health.

## Teaser

This cohort study examines the association between the physical activity levels of women before and during pregnancy and neurodevelopment of their child from 6 months to 3 years of age.

## Introduction

The influence of the health of pregnant women on fetal growth and development is well established. Recently, many studies have shown that high levels of physical activity during pregnancy positively affect the health of both the mother and fetus.^1,2,3,4,5,6,7^ Therefore, moderate exercise during pregnancy has increasingly been recommended.^1,3^

Excluding special conditions such as preexisting maternal diseases, complications during the pregnancy, cervical insufficiency, or threatened miscarriage, moderate exercise in pregnant women without complications helps minimize a decline in physical fitness and improve cardiovascular function; it also contributes to weight control during pregnancy.^4,8^ Moderate exercise during pregnancy contributes to improved quality of sleep, reduced duration and pain in labor, and alleviation of depressive symptoms.^2,6,7^ Recommended levels of physical activity for pregnant women without complications have also been clearly specified.^3,8,9^ Furthermore, exercising during pregnancy helps prevent preterm birth,^1,10^ reduce the risk of obesity, improve neuromotor development in children,^5,11,12,13^ and contribute to the child’s emotional stability.^3,4^ Thus, engaging in physical activity during pregnancy both benefits the mother’s health and, in various aspects, has positive effects on the child to be born. Although the short-term effects of physical activity during pregnancy on the child’s neurodevelopment have been analyzed,^11,12,13,14,15^ large-scale and longitudinal follow-up studies remain scarce.^16^ Moreover, because the assessment tools used in previous reports have not been widely adopted, we considered that conducting research using internationally standardized assessment tools was necessary.

Therefore, in this study, we investigated maternal physical activity levels before and during pregnancy using the data from a large-scale birth cohort study targeting pregnant women and their children in Japan. We examined the association between the physical activity levels of the mother and the neurodevelopment of the child.

## Methods

### Study Design and Population

In this cohort study, we analyzed data from the Japan Environment and Children’s Study (JECS) conducted by the Ministry of the Environment. This nationwide birth cohort study recruited approximately 100 000 mother-child pairs nationwide from January 24, 2011, to March 31, 2014.

**Figure 1.**
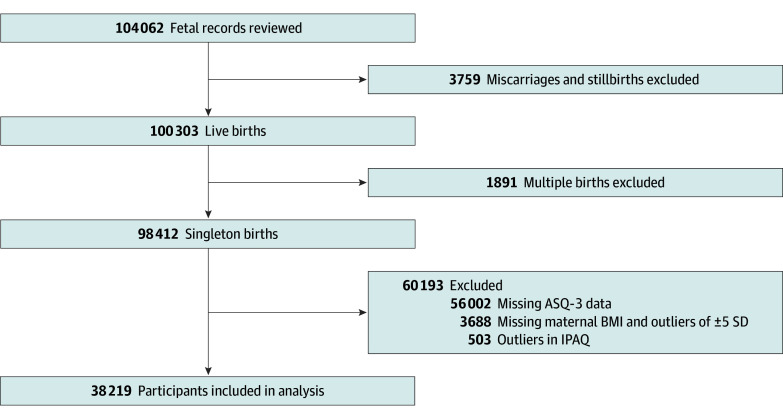
Study Flowchart ASQ-3 indicates Ages and Stages Questionnaire, Third Edition; BMI, body mass index; and IPAQ, International Physical Activity Questionnaire.

Of the total 104 062 fetal records, those for stillbirths, abortions, and multiple fetuses as well as those with missing information on the neurodevelopment of the child from 6 months to 3 years old or in which the maternal body mass index (BMI) value was ±5 SDs were excluded (Figure 1). Outliers in maternal physical activity were also excluded, according to the International Physical Activity Questionnaire (IPAQ) analysis guidelines.^17^ We used the dataset called jecs-ta-20190930 in this study. The protocol of the JECS^18^ was reviewed and approved by the Institutional Review Board on Epidemiological Studies of the Ministry of the Environment and the Ethics Committees of all participating institutions. Written informed consent was obtained from all participants. This study was conducted and reported in accordance with the Strengthening the Reporting of Observational Studies in Epidemiology (STROBE) reporting guideline.

### Maternal Physical Activity

Maternal physical activity levels were assessed using questionnaires administered at 2 time points. The first questionnaire, completed during pregnancy, assessed prepregnancy activity, while the second, completed midpregnancy (16-27 weeks of gestation), assessed activity during that period. Both questionnaires evaluated the typical weekly physical activity of the participants. The short version of the IPAQ was used to assess the physical activity levels of pregnant women. The IPAQ was developed as a standardized tool to assess physical activity levels across diverse populations worldwide.^19,20^ Based on the IPAQ guidelines,^17,19^ the total levels of physical activity in metabolic equivalent (MET) minutes per week were calculated based on the duration of walking, moderate, and vigorous physical activity. These were classified into low, moderate, and high levels of physical activity. In this study, physical activity levels were further classified into 3 categories: none (AL0; MET-min/wk = 0), low (AL1), and moderate to high (AL2). Based on the IPAQ analysis guidelines,^17^ all cases in which the sum of the total time variables of all walking, moderate, and vigorous intensity activities exceeded 960 minutes (16 hours per day) were excluded from the analysis. This exclusion criterion assumes an average sleeping time of 8 hours per day. Responses of less than 10 minutes were reset to zero because continuous exercise for at least 10 minutes is required to achieve health benefits.^21^

### Child Neurodevelopment

The Ages and Stages Questionnaire, Third Edition (ASQ-3), was used to assess child neurodevelopment.^22^ The ASQ-3 is a screening tool for developmental delay designed for children aged 1 to 66 months. It consists of 5 developmental domains: communication, gross motor skills, fine motor skills, problem solving, and personal-social. Each domain contains 6 questions. Parents are asked to select the most appropriate response from 3 options: yes, if their child can do the activity; sometimes, if their child can sometimes do the activity; and not yet, if their child cannot do the activity. A score of 10 points is assigned for yes, 5 points for sometimes, and 0 points for not yet. The total score thus ranges from 0 to 60 for each domain, and the scores are compared with the cutoff values for each domain. Evaluations were conducted at 6 months and 1.0, 1.5, 2.0, 2.5, and 3.0 years of age. Participants who answered all 30 questions across all time points were included in the analysis. The cutoff values used in this study were those reported by Mezawa et al (eTable 1 in Supplement 1).^23^

### Covariates and Measurements

Based on previous studies, we set the covariates influencing child neurodevelopment as gestational age, birth weight, maternal age, prepregnancy BMI, predelivery BMI,^5^ the use of group childcare,^24,25^ number of siblings at midpregnancy,^5,11^ family income at midpregnancy,^26,27^ and the educational backgrounds of the parents.^11,27^ The levels of maternal physical activity were classified into AL0 to AL2 for both prepregnancy and midpregnancy, based on exercise habits and intensity. For each group, maternal age, prepregnancy and predelivery BMI, gestational week, birth weight of the child, and the presence or absence of pregnancy complications were analyzed. Child development was assessed by the percentage of children whose scores fell below the cutoff value.

### Statistical Analysis

Data were analyzed from June 24, 2024, to June 30, 2025. Results were examined using either the χ^2^ test or Fisher exact test, depending on the expected cell counts: the former was applied when all expected counts were 5 or greater, and the latter was used when any expected count was less than 5. Multivariable logistic regression analysis was conducted with each age group and each ASQ-3 category as the dependent variables. Maternal activity levels were included as the main independent variables, along with the covariates listed previously. Sex-stratified analyses were also performed. Large differences in the number of participants were observed across some categories, possibly making the estimates unstable. Therefore, the 2 covariates of household income and parental educational level were grouped into fewer categories to ensure analytical stability and improve interpretability. Household income was reclassified into 3 categories: less than ¥6.00, ¥6.00 to ¥9.99, and ¥10.00 million or more (¥0.012-¥0.013 in 2011 = US $1). The educational backgrounds of parents were recategorized into 3 groups: junior high school and high school; college of technology, vocational school, and junior college (technical or high school); and university and graduate school. This approach was adopted to ensure adequate sample sizes across all groups, thereby yielding more robust estimates in the multivariable models. To evaluate the proportion of children scoring below the cutoff values in each ASQ-3 domain across maternal physical activity levels, 3 pairwise comparisons were performed for each age group (AL0 vs AL1, AL0 vs AL2, and AL1 vs AL2). To control for the increased risk of type I error due to multiple comparisons, the resulting *P* values (raw *P*) were adjusted using the Benjamini-Hochberg method to control the false discovery rate. All statistical analyses were conducted using R, version 4.4.0 (R Project for Statistical Computing). X-sided *P* < .05 was considered statistically significant.

## Results

A total of 38 219 mother-child pairs were included in the analysis (maternal mean [SD] age, 31.1 [4.8] years; 19 429 [50.8%] male and 18 783 [49.1%] female children, with 7 [0.02%] missing). The characteristics of the mothers and their children are presented in Table 1. In the prepregnancy stage, the highest proportion (16 212 [42.4%]) of the 38 219 mothers were categorized with AL2. The activity levels decreased midpregnancy, with a rise in the proportions with AL0 and AL1. The median prepregnancy BMI (calculated as weight in kilograms divided by height in meters squared) was 21.2 (IQR, 19.1-22.4; range, 13.0-37.0). By contrast, the median BMI before delivery was 25.2 (IQR, 23.0-26.8; range, 13.9-40.9). Among the newborns, 36 550 (95.6%) were delivered at full term with 34 916 (91.4%) having a normal birth weight. Preterm births before 37 weeks of gestation accounted for 1580 newborns (4.1%), and a decrease in the proportion of preterm births was associated with an increased level of physical activity midpregnancy. Infants with low birth weight accounted for 2974 newborns (7.8%) and, similarly, a higher level of physical activity midpregnancy was associated with a significant reduction in the incidence of low birth weight.

**Table 1.** Maternal and Child Characteristics and Pregnancy Complications

Characteristic	Maternal physical activity level, No. (%)								
	All (N = 38 219 [100])	Prepregnancy				Midpregnancy			
		AL0 (n = 7189 [18.8%])	AL1 (n = 14 818 [38.8%])	AL2 (n = 16 212 [42.4%])	*P* value	AL0 (n = 10 153 [26.6%])	AL1 (n = 16 774 [43.9%])	AL2 (n = 11 292 [29.5%])	*P* value
**Maternal characteristics**									
Age, y									
<25	3149 (8.2)	580 (8.1)	1024 (6.9)	1545 (9.5)	<.001	738 (7.3)	1443 (8.6)	968 (8.6)	<.001
25-29	10 681 (27.9)	1930 (26.8)	3981 (26.9)	4770 (29.4)		2596 (25.6)	4678 (27.9)	3407 (30.2)	
30-34	13 053 (34.2)	2413 (33.6)	5203 (35.1)	5437 (33.5)		3414 (33.6)	5755 (34.3)	3884 (34.4)	
35-39	7758 (20.3)	1542 (21.4)	3171 (21.4)	3045 (18.8)		2338 (23.0)	3349 (20.0)	2071 (18.3)	
≥40	1340 (3.5)	252 (3.5)	602 (4.1)	486 (3.0)		399 (3.9)	576 (3.4)	365 (3.2)	
Missing data	2238 (5.9)	472 (6.6)	837 (5.6)	929 (5.7)		668 (6.6)	973 (5.8)	597 (5.3)	
Prepregnancy BMI									
<18.5	6185 (16.2)	1246 (17.3)	2401 (16.2)	2538 (15.7)	.009	1660 (16.3)	2730 (16.3)	1795 (15.9)	.76
18.5-24.9	28 171 (73.7)	5274 (73.4)	10 869 (73.3)	12 028 (74.2)		7489 (73.8)	12 324 (73.5)	8358 (74.0)	
25.0-29.9	2927 (7.7)	516 (7.2)	1167 (7.9)	1244 (7.7)		770 (7.6)	1303 (7.8)	854 (7.6)	
≥30.0	767 (2.0)	128 (1.8)	309 (2.1)	330 (2.0)		198 (2.0)	342 (2.0)	227 (2.0)	
Missing data	169 (0.4)	25 (0.3)	72 (0.5)	72 (0.4)		36 (0.4)	75 (0.4)	58 (0.5)	
Predelivery BMI									
<18.5	109 (0.3)	22 (0.3)	33 (0.2)	54 (0.3)	.02	26 (0.3)	45 (0.3)	38 (0.3)	.69
18.5-24.9	19 976 (52.3)	3826 (53.2)	7801 (52.6)	8349 (51.5)		5309 (52.3)	8799 (52.5)	5868 (52.0)	
25.0-29.9	14 719 (38.5)	2735 (38.0)	5657 (38.2)	6327 (39.0)		3924 (38.6)	6422 (38.3)	4373 (38.7)	
≥30.0	2744 (7.2)	474 (6.6)	1061 (7.2)	1209 (7.5)		717 (7.1)	1229 (7.3)	798 (7.1)	
Missing data	671 (1.8)	132 (1.8)	266 (1.8)	273 (1.7)		177 (1.7)	279 (1.7)	215 (1.9)	
**Child characteristics**									
Gestational age, wk									
<37	1580 (4.1)	351 (4.9)	623 (4.2)	606 (3.7)	<.001	559 (5.5)	651 (3.9)	370 (3.3)	<.001
37-41	36 550 (95.6)	6823 (94.9)	14 157 (95.5)	15 570 (96.0)		9575 (94.3)	16 070 (95.8)	10 905 (96.6)	
≥42	83 (0.2)	15 (0.2)	36 (0.2)	32 (0.2)		18 (0.2)	49 (0.3)	16 (0.1)	
Missing data	6 (0.02)	0	2 (0.01)	4 (0.0)		1 (0.01)	4 (0.02)	1 (0.01)	
Birth weight, g									
<2500	2974 (7.8)	590 (8.2)	1133 (7.6)	1251 (7.7)	.29	890 (8.8)	1259 (7.5)	825 (7.3)	<.001
2500-3999	34 916 (91.4)	6540 (91.0)	13 556 (91.5)	14 820 (91.4)		9174 (90.4)	15 379 (91.7)	10 363 (91.8)	
≥4000	323 (0.8)	57 (0.8)	127 (0.9)	139 (0.9)		86 (0.8)	134 (0.8)	103 (0.9)	
Missing data	6 (0.02)	2 (0.03)	2 (0.01)	2 (0.02)		3 (0.03)	2 (0.01)	1 (0.01)	
**Pregnancy complications**									
Threatened preterm labor	7508 (19.6)	1501 (20.9)	2799 (18.9)	3208 (19.8)	.26	2428 (23.9)	2957 (17.6)	2123 (18.8)	<.001
Premature rupture of the membrane	3422 (9.0)	602 (8.4)	1286 (8.7)	1534 (9.5)	.002	871 (8.6)	1489 (8.9)	1062 (9.4)	.003
Placenta previa	236 (0.6)	53 (0.7)	100 (0.7)	83 (0.5)	.02	94 (0.9)	90 (0.5)	52 (0.5)	<.001
Placental abruption	152 (0.4)	24 (0.3)	66 (0.4)	62 (0.4)	.81	53 (0.5)	64 (0.4)	35 (0.3)	.01

Abbreviations: AL0, no physical activity; AL1, low physical activity level; AL2, high physical activity level; BMI, body mass index (calculated as weight in kilograms divided by height in meters squared).

Mothers who engaged in physical activity midpregnancy had a significantly reduced risk of threatened preterm labor (2428 of 10 153 [23.9%] for AL0 vs 2123 of 11 292 [18.8%] for AL2; *P* < .001) and placental abruption (53 of 10 153 [0.5%] for AL0 vs 35 of 11 292 [0.3%] for AL2; *P* = .01). A slight but significant reduction in the incidence of placenta previa was observed with prepregnancy (53 of 7189 [0.7%] for AL0 vs 83 of 16 212 [0.5%] for AL2; *P* = .02) and midpregnancy (94 of 10 153 [0.9%] for AL0 vs 52 of 11 292 [0.5%] for AL2; *P* < .001) maternal physical activity. Higher levels of physical activity were associated with an increased incidence of premature rupture of the membranes prepregnancy (602 of 7189 [8.4%] for AL0 vs 1534 of 16 212 [9.5%] for AL2; *P* = .002) and midpregnancy (871 of 10 153 [8.6%] for AL0 vs 1062 of 11 292 [9.4%] for AL2; *P* = .003), although the difference levels of each group appeared small. No significant differences in the incidence of maternal complications were observed, regardless of the physical activity levels (eTable 2 in Supplement 1).

The covariates considered to be associated with the ASQ-3 scores^11,24,25,26,27^ are presented in Table 2. At 6 months of age, 2734 children (7.2%) attended group childcare, compared with 24 130 (61.5%) by 3 years of age. Approximately half of the children had siblings. Approximately 80% of family incomes were classified as low to middle. The distributions of group childcare attendance at other ages, number of siblings, detailed family income, and parental educational level are presented in eTable 3 in Supplement 1.

**Table 2.** Covariates and Maternal Physical Activity

Covariate	Maternal physical activity, No. (%) of participants					
	Prepregnancy			Midpregnancy		
	AL0 (n = 7189 [18.8%])	AL1 (n = 14 818 [38.8%])	AL2 (n = 16 212 [42.4%])	AL0 (n = 10 153 [26.6%])	AL1 (n = 16 774 [43.9%])	AL2 (n = 11 292 [29.5%])
Group childcare at age 6 mo						
Attending	601 (8.4)	901 (6.1)	1232 (7.6)	817 (8.0)	945 (5.6)	972 (8.6)
Not attending	6588 (91.6)	13 917 (93.9)	14 980 (92.4)	9336 (92.0)	15 829 (94.4)	10 320 (91.4)
Group childcare at age 3 y						
Attending	4851 (67.5)	8833 (59.6)	10 446 (64.4)	6886 (67.8)	9848 (58.7)	7396 (65.5)
Not attending	2338 (32.5)	5985 (40.4)	5766 (35.6)	3267 (32.2)	6926 (41.3)	3896 (34.5)
Presence of siblings						
Yes	3337 (46.4)	7804 (52.7)	8009 (49.4)	5255 (51.8)	8122 (48.4)	5773 (51.1)
None	3852 (53.6)	7014 (47.3)	8203 (50.6)	4898 (48.2)	8652 (51.6)	5519 (48.9)
Family income, ¥^a^						
<6.00 Million	4744 (66.0)	10 107 (68.2)	10 975 (67.7)	6529 (64.3)	11 793 (70.3)	7504 (66.5)
6.00 to 9.99 Million	1644 (22.9)	3323 (22.4)	3621 (22.3)	2452 (24.2)	3433 (20.5)	2703 (23.9)
≥10.00 Million	264 (3.7)	630 (4.3)	695 (4.3)	424 (4.2)	650 (3.9)	515 (4.6)
Missing data	537 (7.5)	758 (5.1)	921 (5.7)	748 (7.4)	898 (5.4)	570 (5.0)
Mother’s educational level						
Junior high school or high school	2567 (35.7)	4546 (30.7)	5080 (31.3)	3364 (33.1)	5383 (32.1)	3446 (30.5)
College of technology, vocational school, or junior college	3032 (42.2)	6291 (42.5)	7208 (44.5)	4341 (42.8)	7094 (42.3)	5096 (45.1)
University or graduate school	1496 (20.8)	3905 (26.4)	3839 (23.7)	2258 (22.2)	4265 (25.4)	2717 (24.1)
Missing data	94 (1.3)	76 (0.5)	85 (0.5)	190 (1.9)	32 (0.2)	33 (0.3)
Father’s educational level						
Junior high school or high school	3187 (44.3)	5789 (39.1)	6640 (40.9)	4340 (42.7)	6689 (39.9)	4587 (40.6)
College of technology, vocational school, or junior college	1664 (23.1)	3265 (22.0)	3770 (23.3)	2297 (22.6)	3783 (22.6)	2619 (23.2)
University or graduate school	2211 (30.8)	5639 (38.1)	5646 (34.8)	3288 (32.4)	6212 (37.0)	3996 (35.4)
Missing data	127 (1.8)	125 (0.8)	156 (1.0)	228 (2.2)	90 (0.5)	90 (0.8)

Abbreviations: AL0, no physical activity level; AL1, low physical activity level; AL2, high physical activity level.

^a^ To convert Japanese yen to 2026 US dollars, multiply by 0.0064.

The results of the multivariable logistic regression analysis of the ASQ-3 at 6 months and 3 years of age are presented in Table 3. Among children 6 months of age, having mothers in the prepregnancy AL1 category was associated with higher odds of scoring above the ASQ-3 cutoff value only in the domain of gross motor skills (odds ratio [OR], 1.12; 95% CI, 1.00-1.25). The category of prepregnancy AL2 showed associations across all ASQ-3 domains, with particularly higher odds observed in the communication (OR vs AL0, 1.61; 95% CI, 1.04-2.45), gross motor (OR vs AL0, 1.37; 95% CI, 1.22-1.54), and fine motor (OR vs AL0, 1.32; 95% CI, 1.13-1.54) domains. In contrast, participation in group childcare and having a sibling were associated with lower odds in some domains. Both midpregnancy AL1 and AL2 were associated with significantly higher odds in the gross motor (AL1 vs AL0 OR, 1.11 [95% CI, 1.00-1.23]; AL2 vs AL0 OR, 1.18 [95% CI, 1.06-1.33]), fine motor (AL1 vs AL0 OR, 1.24 [95% CI, 1.09-1.41]; AL2 vs AL0 OR, 1.60 [95% CI, 1.37-1.86]), and problem solving (AL1 vs AL0 OR, 1.15 [95% CI, 1.04-1.27]; AL2 vs AL0 OR, 1.23 [95% CI, 1.10-1.38]) domains. At 3 years of age, maternal physical activity was associated only with problem solving in the prepregnancy AL2 category (OR, 1.16; 95% CI, 1.01-1.34). Participation in group childcare showed particularly high odds in the communication and personal-social domains, while having a sibling showed particularly high odds in the gross and fine motor domains. At both 6 months and 3 years of age, sex-stratified analyses showed no substantial differences from the overall trends (eTables 4 and 5 in Supplement 1). At 6 months of age, the OR for gestational age was as high as that for maternal exercise and was significant in almost all domains. Many factors, such as family income and parental education, showed strong associations with each domain, which confirmed the reproducibility of a previous report based on the JECS.^24^

**Table 3.** Results of Multivariate Analysis for Each ASQ-3 Domain

Covariate	ASQ-3 domain									
	Communication		Gross motor		Fine motor		Problem solving		Personal-social	
	OR (95% CI)	*P* value	OR (95% CI)	*P* value	OR (95% CI)	*P* value	OR (95% CI)	*P* value	OR (95% CI)	*P* value
**Age 6 mo**										
Prepregnancy										
AL0	1 [Reference]	NA	1 [Reference]	NA	1 [Reference]	NA	1 [Reference]	NA	1 [Reference]	NA
AL1	1.21 (0.80-1.81)	.34	1.12 (1.00-1.25)	.04	1.09 (0.94-1.27)	.24	1.05 (0.94-1.17)	.37	1.05 (0.88-1.26)	.54
AL2	1.61 (1.04-2.45)	.02	1.37 (1.22-1.54)	<.001	1.32 (1.13-1.54)	<.001	1.18 (1.05-1.32)	.003	1.24 (1.03-1.48)	.01
Group childcare	1.24 (0.64-2.78)	.55	0.98 (0.82-1.17)	.85	1.10 (0.87-1.41)	.43	0.84 (0.71-0.99)	.03	0.77 (0.61-1.00)	.04
Siblings	0.87 (0.73-1.04)	.12	0.91 (0.86-0.95)	<.001	0.90 (0.84-0.96)	.002	0.96 (0.91-1.01)	.11	0.85 (0.79-0.92)	<.001
Midpregnancy										
AL0	1 [Reference]	NA	1 [Reference]	NA	1 [Reference]	NA	1 [Reference]	NA	1 [Reference]	NA
AL1	1.42 (0.97-2.07)	.07	1.11 (1.00-1.23)	.03	1.24 (1.09-1.41)	.001	1.15 (1.04-1.27)	.004	1.14 (0.98-1.34)	.09
AL2	1.28 (0.85-1.93)	.23	1.18 (1.06-1.33)	.002	1.60 (1.37-1.86)	<.001	1.23 (1.10-1.38)	<.001	1.17 (0.98-1.39)	.07
Group childcare	1.25 (0.65-2.80)	.54	0.98 (0.82-1.17)	.83	1.09 (0.86-1.40)	.46	0.84 (0.71-0.99)	.03	0.77 (0.61-1.00)	.04
Siblings	0.87 (0.73-1.05)	.14	0.91 (0.86-0.96)	<.001	0.90 (0.84-0.96)	.003	0.96 (0.91-1.01)	.14	0.85 (0.79-0.92)	<.001
**Age 3 y**										
Prepregnancy										
AL0	1 [Reference]	NA	1 [Reference]	NA	1 [Reference]	NA	1 [Reference]	NA	1 [Reference]	NA
AL1	1.03 (0.86-1.24)	.69	1.00 (0.83-1.19)	1.00	1.12 (0.97-1.29)	.10	1.11 (0.97-1.28)	.11	.96 (0.78-1.18)	.76
AL2	1.02 (0.85-1.22)	.78	1.10 (0.92-1.31)	.27	1.07 (0.94-1.23)	.28	1.16 (1.01-1.34)	.02	.97 (0.79-1.19)	.82
Group childcare	1.62 (1.42-1.86)	<.001	1.03 (0.90-1.18)	.57	1.07 (0.97-1.19)	.16	1.30 (1.17-1.44)	<.001	1.80 (1.55-2.08)	<.001
Siblings	1.00 (0.92-1.08)	.94	1.33 (1.22-1.45)	<.001	1.21 (1.13-1.29)	<.001	1.06 (1.00-1.13)	.03	1.13 (1.04-1.25)	.005
Midpregnancy										
AL0	1 [Reference]	NA	1 [Reference]	NA	1 [Reference]	NA	1 [Reference]	NA	1 [Reference]	NA
AL1	0.95 (0.80-1.12)	.56	1.02 (0.88-1.19)	.73	1.08 (0.95-1.22)	.20	1.09 (0.97-1.24)	.13	1.02 (0.85-1.22)	.78
AL2	0.92 (0.77-1.09)	.36	1.13 (0.95-1.34)	.15	1.12 (0.98-1.28)	.07	1.13 (0.99-1.30)	.06	0.95 (0.79-1.16)	.67
Group childcare	1.62 (1.41-1.85)	<.001	1.04 (0.91-1.19)	.55	1.07 (0.96-1.19)	.17	1.30 (1.17-1.44)	<.001	1.80 (1.56-2.09)	<.001
Siblings	1.00 (0.92-1.08)	.93	1.33 (1.22-1.45)	<.001	1.21 (1.13-1.29)	<.001	1.07 (1.00-1.14)	.03	1.14 (1.04-1.25)	.004

Abbreviations: AL0, no physical activity level; AL1, low physical activity level; AL2, high physical activity level; OR, odds ratio.

**Figure 2.**
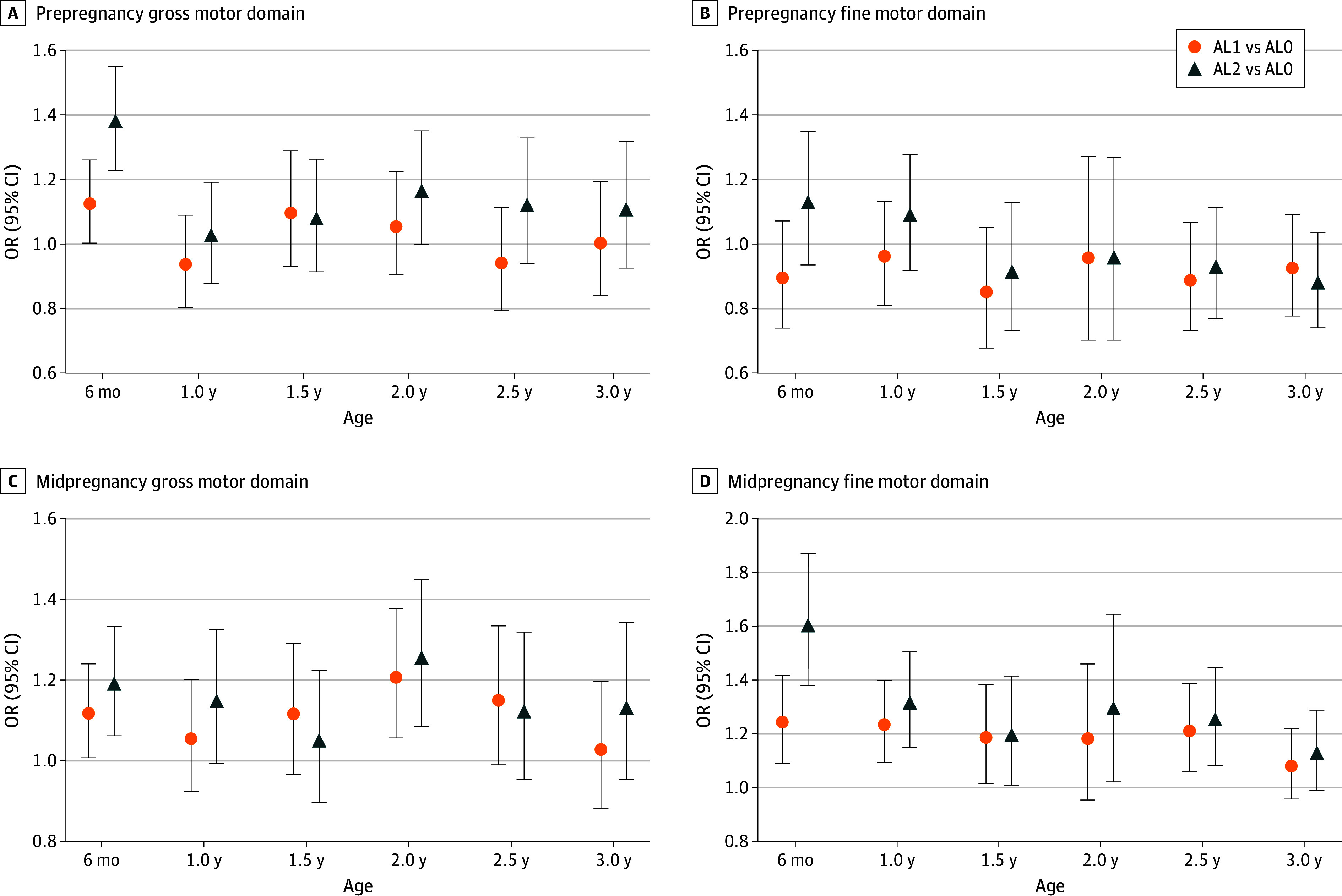
Changes in Ages and Stages Questionnaire Odds Ratios (ORs) by Domain, Age, and Maternal Physical Activity Level ORs were estimated using multivariable logistic regression models adjusted for gestational age, birth weight, maternal age, prepregnancy and predelivery body mass index, maternal physical activity (before or during pregnancy), group childcare attendance, presence of siblings, family income, mother’s education, and father’s education. AL0 indicates no physical activity level; AL1, low physical activity level; and AL2, high physical activity level.

The changes in ORs for the gross and fine motor domains, which showed particularly high values in the multivariable analysis, are illustrated in Figure 2. In the prepregnancy period (Figure 2A and B), ORs at 6 months were markedly higher for both gross (OR vs AL0, 1.37; 95% CI, 1.22-1.54) and fine (OR vs AL0, 1.32; 95% CI, 1.13-1.54) motor skills in the AL2 category. In the fine motor domain, high ORs persisted at 1 year for both AL1 (OR vs AL0, 1.16; 95% CI, 1.01-1.33) and AL2 (OR vs AL0, 1.28; 95% CI, 1.11-1.47). In midpregnancy (Figure 2C and D), gross motor skills showed associations for AL1 and AL2 at 6 months (ORs vs AL0, 1.11 [95% CI, 1.00-1.23] and 1.18 [95% CI, 1.06-1.33], respectively) and 2 years (ORs vs AL0, 1.20 [95% CI, 1.05-1.37] and 1.25 [95% CI, 1.08-1.44], respectively). Fine motor skills were significant at all ages except 2 and 3 years, regardless of ActLv category, but ORs tended to decrease with increasing age. The trajectories of the 3 domains other than gross and fine motor skills are presented in eFigures 1 and 2 in Supplement 2. Problem solving showed associations from 6 months to 2 years of age, whereas communication and personal-social domains showed associations only at younger ages.

eFigures 3 and 4 in Supplement 2 present the proportion below the cutoff value for each ASQ-3 domain at each age, along with the statistically significant differences between the levels of physical activity. Across nearly all ages and ASQ-3 domains, higher levels of physical activity were associated with a lower proportion below the cutoff value. Statistically significant differences were most evident between AL0 and AL2 categories across nearly all domains and age groups, including gross motor skills at 6 months in prepregnancy and fine motor skills at 6 months in midpregnancy. Significant differences were also found between categories AL1 and AL2 (eg, communication skills at 2 years in prepregnancy and problem solving at 3 years in midpregnancy), as well as between AL0 and AL1 (eg, problem solving at 1.5 years in prepregnancy and fine motor skills at 6 months in midpregnancy) at several time points. Differences between the ActLv categories were generally clearer and significant midpregnancy compared with prepregnancy across all ASQ-3 domains.

## Discussion

The present study, which extended the follow-up period of a previous study involving the same cohort,^13^ focused on the time course of child neurodevelopment until 3 years of age. New evidence is presented that a mother’s physical activity habits may be associated with their child’s neurodevelopment from infancy through early childhood. Motor development showed particularly strong associations at the age of 6 months and at 1 year.

The factors that affect the children’s neurodevelopmental behavior remain unclear.^16^ However, studies have found that moderate aerobic exercise during pregnancy may promote child neurodevelopment by reducing maternal inflammatory cytokines and stimulating fetal sensory systems, such as vestibular function,^13^ as well as by having beneficial effects on the neuroelectric response of the newborn’s brain.^28^ Brain-derived neurotrophic factor, known to promote brain development and neurogenesis, is also a potential candidate.^16^ In this study, it was also difficult to directly demonstrate how maternal exercise habits during pregnancy relate to fetal responses associated with child neurodevelopment, particularly motor function. However, physical activity during pregnancy helps reduce vascular resistance, which in turn increases blood flow through the umbilical cord connecting the mother and fetus.^3^ Consequently, the fetus receives an adequate supply of blood, supporting its growth and development. Moreover, prepregnancy fitness likely differs between active and sedentary mothers. Better maternal fitness may facilitate smoother labor, potentially enhancing child neurodevelopment. The present study also suggested a clear association between group childcare and neurodevelopment, consistent with previous studies.^24,25^ Child neurodevelopment is multifactorial. Our findings suggest that maternal exercise optimizes the intrauterine environment and fetal stimuli, enhancing motor function in early infancy (6-12 months of age). However, this prenatal influence may diminish with age as environmental factors,^15,29^ such as group childcare, become more dominant. Specifically, factors related to the home environment—such as family income,^26^ parental educational levels,^27^ media exposure,^30^ and sleep duration,^31^—are likely to play a larger role. The possibility of emerging epigenetic differences has also been considered.^32,33^ The diminishing influence of maternal factors with the child’s age was discussed in a previous study^13^ and stated that longer-term investigation would be necessary to confirm the diminishing influence of these factors.

Generally, exercise during pregnancy is only recommended for women with no complications. Maternal rest is crucial in the presence of risk factors for preterm birth, such as cervical insufficiency or a shortened cervix.^3^ In the present study, the proportion of participants in the midpregnancy AL0 category was elevated in cases of threatened preterm labor. Furthermore, higher levels of midpregnancy physical activity were associated with a reduced risk of threatened preterm labor. This may reflect an increase in the number of pregnant women who were instructed to rest due to signs of threatened preterm labor. However, the present findings indicate that a higher level of midpregnancy physical activity was associated with a lower rate of preterm birth, suggesting that moderate exercise in pregnant women without complications may contribute to the prevention of preterm birth. This may suggest, as noted in previous studies,^3,7^ that moderate physical activity helps keep pregnant women mentally and physically healthy.

### Limitations 

This study has some limitations. First, selection bias is a concern. The requirement to complete numerous questionnaires may have recruited more health-conscious participants. Furthermore, each ASQ-3 questionnaire included 30 items (6 items per domain across 5 domains) and was administered at 0.5, 1.0, 1.5, 2.0, 2.5, and 3.0 years of age. Participants with any missing response at any time point were excluded; thus, only those with complete data across all assessment waves were included. Excluding those with missing ASQ-3 data at any time point reduced the original sample by more than half, potentially selecting for highly engaged participants with higher socioeconomic status.

Second, maternal physical activity levels may be imprecise. Reliance on self-reported questionnaires allows subjective interpretation and recall bias, potentially overestimating or underestimating exercise intensity and duration. Third, reverse causation cannot be ruled out. Mothers with pregnancy complications or threatened preterm labor may have reduced their activity due to medical advice, complicating the determination of causality. Last, the generalizability of these findings may be limited to populations with similar socioeconomic and cultural backgrounds.

## Conclusions

In this cohort study, maternal physical activity before and during pregnancy was associated with child neurodevelopment. Observed odds were significantly greater for motor function between 6 months and 1 year of age. Further investigations are required to find the physiological mechanisms explaining how maternal physical activity affects child neurodevelopment.

## Notes

Supplement 1. **eTable 1.** Cutoff Values of the ASQ-3 **eTable 2.** Characteristics of Maternal Complications **eTable 3.** Characteristics of Covariates and Maternal Physical Activity **eTable 4.** Results of Multivariate Analysis for Each ASQ-3 Domain at 6 Months of Age **eTable 5.** Results of Multivariate Analysis for Each ASQ-3 Domain at 3 Years of Age

Supplement 2. **eFigure 1. **Prepregnancy Changes in ASQ-3 Odds Ratios by Domain, Age, and Maternal Physical Activity Level **eFigure 2. **Midpregnancy Changes in ASQ-3 Odds Ratios by Domain, Age, and Maternal Physical Activity Level **eFigure 3. **Prepregnancy Proportion of Children Below the ASQ-3 Cutoff by Domain, Age, and Maternal Physical Activity Level **eFigure 4. **Midpregnancy Proportion of Children Below the ASQ-3 Cutoff by Domain, Age, and Maternal Physical Activity Level

Supplement 3.
Nonauthor Collaborators

Supplement 4.
Data Sharing Statement
