# Supplementary material for: Physical Activity Before and During Pregnancy and Neurodevelopment in Early Childhood
Source: JAMA Netw Open. 2026 Mar 3;9(3):e260345. doi: 10.1001/jamanetworkopen.2026.0345 (PMC12958087; doi:10.1001/jamanetworkopen.2026.0345)
Supplement: Supplement 2. — eFigure 1. Prepregnancy Changes in ASQ-3 Odds Ratios by Domain, Age, and Maternal Physical Activity Level eFigure 2. Midpregnancy Changes in ASQ-3 Odds Ratios by Domain, Age, and Maternal Physical Activity Level eFigure 3. Prepregnancy Proportion of Children Below the ASQ-3 Cutoff by Domain, Age, and Maternal Physical Activity Level eFigure 4. Midpregnancy Proportion of Children Below the ASQ-3 Cutoff by Domain, Age, and Maternal Physical Activity Level [file jamanetwopen-e260345-s002.pdf]

Supplementary Online Content

Kumasaka I, Suzuki T, Kanamori K, Miura Y, Ota C, Japan Environment and Children's Study Group. Physical activity before and during pregnancy and neurodevelopment in early childhood. *JAMA Netw Open.* 2026;9(3):e260345. doi:10.1001/jamanetworkopen.2026.0345

eFigure 1. Prepregnancy Changes in ASQ-3 Odds Ratios by Domain, Age, and Maternal Physical Activity Level

eFigure 2. Midpregnancy Changes in ASQ-3 Odds Ratios by Domain, Age, and Maternal Physical Activity Level

eFigure 3. Prepregnancy Proportion of Children Below the ASQ-3 Cutoff by Domain, Age, and Maternal Physical Activity Level

eFigure 4. Midpregnancy Proportion of Children Below the ASQ-3 Cutoff by Domain, Age, and Maternal Physical Activity Level

This supplementary material has been provided by the authors to give readers additional information about their work.

© 2026 Kumasaka I et al. *JAMA Network Open*.

eFigure 1. Prepregnancy Changes in ASQ-3 Odds Ratios by Domain, Age, and Maternal Physical Activity Level

This figure shows the trends in the odds ratios for the three ASQ-3 domains that are not presented in the main text.

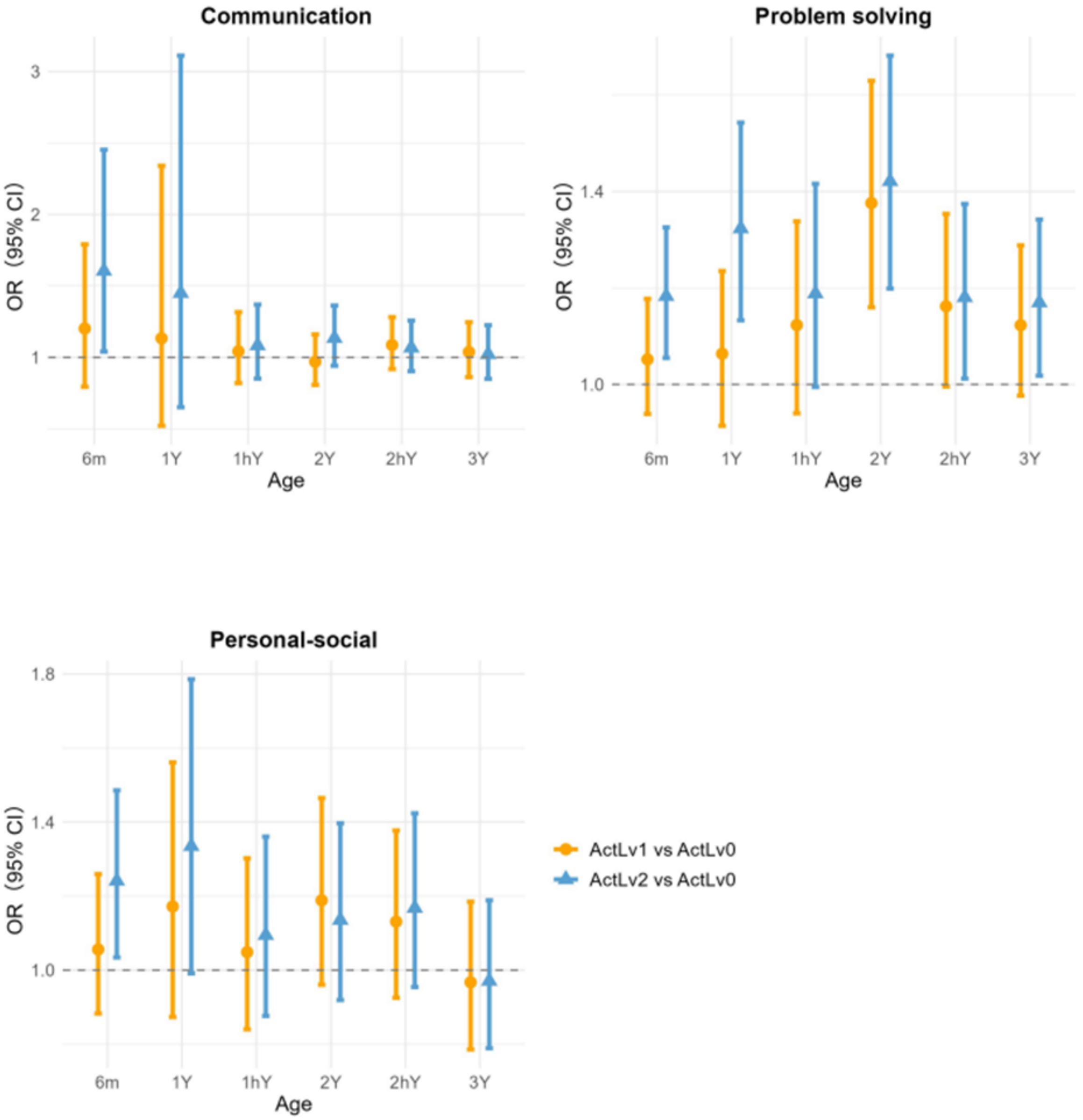

© 2026 Kumasaka I et al. *JAMA Network Open*.

eFigure 2. Midpregnancy Changes in ASQ-3 Odds Ratios by Domain, Age, and Maternal Physical Activity Level

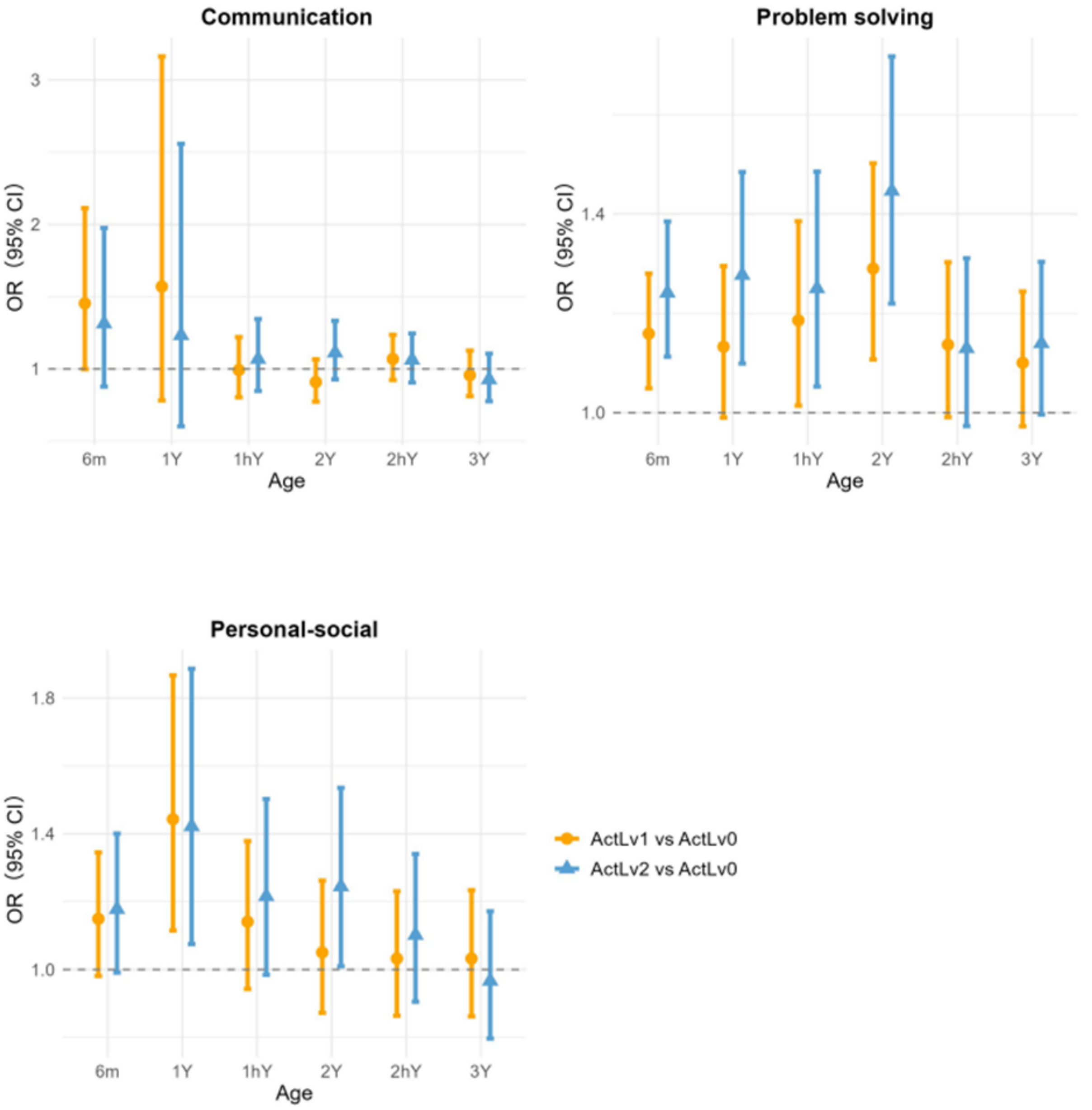

© 2026 Kumasaka I et al. *JAMA Network Open*.

eFigure 3. Prepregnancy Proportion of Children Below the ASQ-3 Cutoff by Domain, Age, and Maternal Physical Activity Level

This figure shows the proportion of children scoring below the cutoff value for each ASQ-3 domain by age, stratified by maternal physical activity level. The results indicate that higher maternal physical activity is associated with a lower proportion of children scoring below the cutoff value.

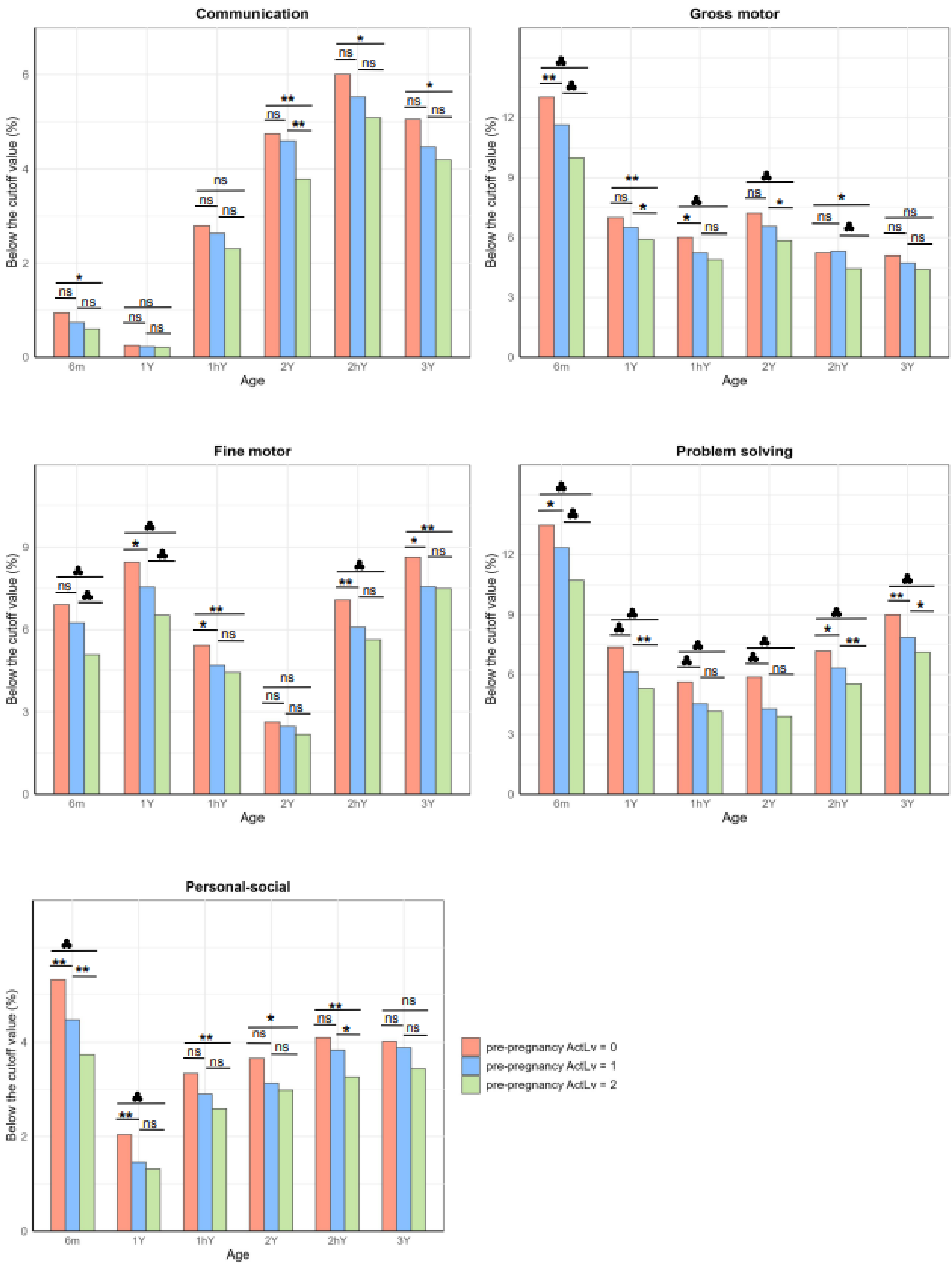

© 2026 Kumasaka I et al. *JAMA Network Open*.

eFigure 4. Midpregnancy Proportion of Children Below the ASQ-3 Cutoff by Domain, Age, and Maternal Physical Activity Level

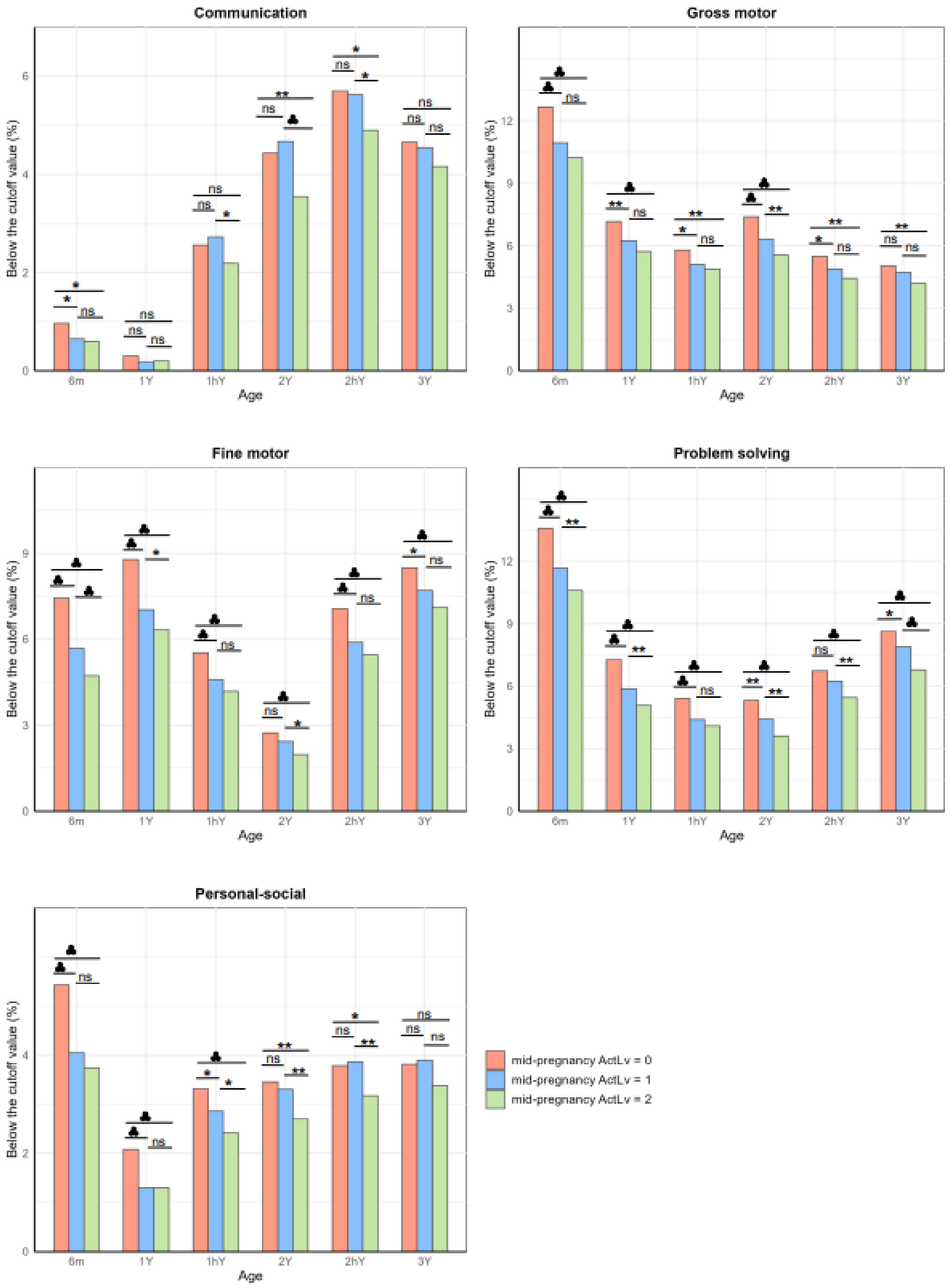

ActLv0: no physical activity level; ActLv1: low physical activity level; ActLv2: high physical activity level; ns: not significant; *: p < 0.05; **: p < 0.01; ⁂: p < 0.001

© 2026 Kumasaka I et al. *JAMA Network Open*.
