# Supplementary material for: Physical Activity Before and During Pregnancy and Neurodevelopment in Early Childhood
Source: JAMA Netw Open. 2026 Mar 3;9(3):e260345. doi: 10.1001/jamanetworkopen.2026.0345 (PMC12958087; doi:10.1001/jamanetworkopen.2026.0345)
Supplement: Supplement 3. — Nonauthor Collaborators [file jamanetwopen-e260345-s003.pdf]

Supplemental Online Content: Nonauthor Collaborators
*First name, last name, and suffix (if applicable) are required and will appear in PubMed.

*Group Name(s): Japan Environment and Children's Study Group								
***First Name and Middle Initial(s)+A2:H15**	***Last Name**	***Suffix (eg, Jr, III)**	Academic Degrees	Institution	Location (city, state/province, country)	Role or Contribution, eg, chair, principal investigator	Group (if more than 1 Group listed in the byline) and/or Subgroup (eg, Steering Committee)	
Michihiro	Kamijima		MD, PhD	Nagoya City University	Nagoya, Japan	Principal Investigato	Aichi Regional Center	
Shin	Yamazaki		DrPH	National Institute for Environmental Studies	Tsukuba, Japan		Programme Office	
Maki	Fukami		MD, PhD	National Center for Child Health and Development	Tokyo, Japan		Medical Support Centre	
Reiko	Kishi		MD, PhD, MPH	Hokkaido University	Sapporo, Japan		Hokkaido Regional Centre	
Koichi	Hashimoto		MD, PhD	Fukushima Medical University	Fukushima, Japan		Fukushima Regional Center	
Kenichi	Sakurai		PhD	Chiba University	Chiba, Japan		Chiba Regional Centre	
Shuichi	Ito		MD, PhD	Yokohama City University	Yokohama, Japan		Kanagawa Regional Centre	
Ryoji	Shinohara		PhD	University of Yamanashi	Chuo, Japan		Koshin Regional Centre	
Hidekuni	Inadera		MD, PhD	University of Toyama	Toyama, Japan		Toyama Regional Centre	
Takeo	Nakayama		MD, PhD	Kyoto University	Kyoto, Japan		Kyoto Regional Centre	
Ryo	Kawasaki		MD, PhD, MPH	Osaka University	Suita, Japan		Osaka Regional Centre	
Yasuhiro	Takeshima		MD, PhD	Hyogo Medical University	Nishinomiya, Japan		Hyogo Regional Centre	
Hideki	Nagashima		MD, PhD	Tottori University	Yonago, Japan		Tottori Regional Centre	
Narufumi	Suganuma		MD, PhD	Kochi University	Nankoku, Japan		Kochi Regional Centre	
Mayumi	Tsuji		MD, PhD	University of Occupational and Environmental Health	Kitakyushu, Japan		Fukuoka Regional Centre	
Kimitoshi	Nakamura		MD, PhD	Kumamoto University	Kumamoto, Japan		South Kyusyu/Okinawa Regional Centre	
