# Supplementary material for: Physical Activity Before and During Pregnancy and Neurodevelopment in Early Childhood
Source: JAMA Netw Open. 2026 Mar 3;9(3):e260345. doi: 10.1001/jamanetworkopen.2026.0345 (PMC12958087; doi:10.1001/jamanetworkopen.2026.0345)
Supplement: Supplement 4. — Data Sharing Statement [file jamanetwopen-e260345-s004.pdf]

Data Sharing Statement

Kumasaka. Physical Activity Before and During Pregnancy and Neurodevelopment in Early Childhood. *JAMA Netw Open*. Published March 03, 2026.
doi:10.1001/jamanetworkopen.2026.0345

Data
Data available: No
